# Bta-miR-223 Targeting the RHOB Gene in Dairy Cows Attenuates LPS-Induced Inflammatory Responses in Mammary Epithelial Cells

**DOI:** 10.3390/cells11193144

**Published:** 2022-10-06

**Authors:** Peng Jiao, Jinpeng Wang, Jian Yang, Xingping Wang, Zhuoma Luoreng

**Affiliations:** 1 School of Agriculture, Ningxia University, Yinchuan 750021, China; jp18055238569@126.com (P.J.); 13309402775@126.com (J.W.); yangjian9603@163.com (J.Y.); wxp@nxu.edu.cn (X.W.); 2 Key Laboratory of Ruminant Molecular Cell Breeding, Ningxia Hui Autonomous Region, Yinchuan 750021, China

**Keywords:** bta-miR-223, RHOB, NF-κB, mastitis

## Abstract

Bovine mammary epithelial cells (bMECs) are part of the first line of defense against pathogens. In recent studies, bta-miR-223 has been reported to activate congenital and innate immunity against inflammatory damage during the pathogenesis of mastitis in dairy cows. The purpose of this study was to identify the regulatory mechanism of bta-miR-223 and its downstream target genes in inflammatory bMECs. A double luciferase reporter gene assay demonstrated that ras homolog family member B (RHOB) was the target gene of bta-miR-223. To further elucidate the role of bta-miR-223 in congenital immune responses, bta-miR-223 mimics (mimic/inhibitor) were transfected into bMECs stimulated with lipopolysaccharide (LPS), which activates the Toll-like receptor 4/nuclear factor-κB (TLR4/NF-κB) signaling pathway. Real-time quantitative PCR (qPCR) and Western blot were used to detect the expression of related genes and proteins, and enzyme-linked immunosorbent assay (ELISA) was used to detect secreted inflammatory factors. Results showed that bta-miR-223 expression during inflammation in bMECs reduced the secretion of inflammatory factors by targeting RHOB and deactivation of NF-κB gene activity. Silencing RHOB inhibited LPS-induced inflammatory response in bMECs. Overall, bta-miR-223 attenuated LPS-induced inflammatory response, and acted as a negative feedback regulator via targeting RHOB, providing a novel avenue for mastitis treatment.

## 1. Introduction

Mastitis in dairy cows is an inflammatory disease caused by pathogenic microorganisms infecting the mammary tissue that has a high incidence, low cure rate, frequent recurrence and expensive treatment. Furthermore, mastitis results in decreased milk production, lower milk quality, and even premature culling of cows, causing great economic losses to the dairy industry [1]. In recent years, a substantial body of research has been published on the molecular regulation mechanism of mastitis in dairy cows. The studies demonstrated that the occurrence, development, susceptibility and resistance to mastitis are regulated by a gene network composed of several genes, among which the Toll-like receptor 4/nuclear factor-κB (TLR4/NF-κB) signaling pathway is an important regulatory pathway [2,3]. Lipopolysaccharide (LPS) is a component of the outer membrane of Gram-negative bacteria and is a well-established agonist of TLR4 on the cell membrane surface of host cells, ultimately activating the NF-κB signaling pathway [4,5]. Therefore, the use of LPS stimulation to establish animal models of inflammation has been tested in a variety of species. Stimulation of bovine mammary epithelial cells (bMECs) with LPS significantly increases the expression of inflammatory and chemokine genes, such as interleukin-8 (IL-8), interleukin-6 (IL-6) and interleukin-1β (IL-1β) [6,7].

MicroRNAs (miRNAs) are a class of endogenous non-coding RNAs that regulate intrinsic and adaptive immunity by targeting and inhibiting the 3′ untranslated regions (3′ UTRs) of mRNAs. miR-223 was first identified by qPCR in 2003 [8]. It was previously reported that bta-miR-223 could alleviate inflammation-mediated damage in bMECs by targeting CBLB to inhibit the PI3K/AKT/NF-κB pathway, thereby suppressing IL-6 expression [9]. The functional SNP in the 3′ UTR of the HMGB1 gene affects bta-miR-223 binding, thereby tempering the regulation of mastitis in dairy cows [10]. In recent years, the regulation of RHOB by miR-223 has been reported in other species [11,12,13,14,15,16]. A typical example is that, in LPS-induced A549 (lung adenocarcinoma cells), low expression of miR-223 made targeting RHOB to inhibit the NLRP3 inflammasome and TLR4/NF-κB signaling pathway less effective. This resulted in an exacerbation of lung inflammation-mediated damage [11]. Therefore, miR-223 plays an important regulatory role in the biological processes of inflammatory diseases. However, the molecular regulatory mechanisms in bovine mastitis are not yet clear. Moreover, no molecular regulatory mechanism between bta-miR-223 and RHOB has been identified in cattle at the time of publication. In this study, the molecular regulatory mechanisms of bta-miR-223 on RHOB/NF-κB were explored at the cellular level to elucidate their possible response mechanisms in LPS-treated bMECs.

## 2. Materials and Methods

### 2.1. Cell Culture

The bMECs (an immortalized MAC-T cell line) were cultured in DMEM/F12 medium (Ge Healthcare Life Sciences Hyclone Laboratories, South Logan, Utah) containing 10% fetal bovine serum (FBS) (Biological Industries Israel Beit Haemek Ltdkibbutz Beit Haemek, Israel) [17]. The purity of the bMECs was assessed by immunofluorescence staining to detect the expression of Cytokeratin 18 (a marker protein for epithelial cells) when the bMECs reached 80–90% confluence based on previously reported methods [17]. Human embryonic kidney (HEK)-293T cells were recovered and cultured in high-glucose DMEM (Ge Healthcare Life Sciences Hyclone Laboratories, South Logan, Utah) containing 5% FBS (Biological Industries Israel Beit Haemek Ltdkibbutz Beit Haemek, Israel). All incubation conditions were 37 °C and 5% CO_2_ concentration.

### 2.2. miRNA Mimic, Inhibitors and siRNA

The mimic of miR-223 (dsRNA oligonucleotides), mismatched negative control mimic (NC_mimic), miR-223 inhibitor (single-stranded oligonucleotides) and mismatched negative control inhibitor (NC_inhibitor), each Cy3-labeled NC mimic/inhibitor, were synthesized by RIBOBIO Co., Ltd. (Guangzhou, China). Mimics and inhibitors were used to assess the effects of overexpression and inhibition of miR-223 on its activity in HEK-293 or bMECs. Then, the fluorescence transfection efficiency was measured (Figure S1A,B).

The interference sequence of RHOB was synthesized by Sangon Biotech (Shanghai) Co., Ltd. (siRNA-RHOB labeled with the fluorescent dye FAM, herein referred to as FAM NC), was used to track transfection efficiency (Figure S1C). The sequence of siRNA-RHOB and FAM-labeled NC primers are presented in Table S1.

### 2.3. Dual-Luciferase Reporter Assays

RHOB was predicted to be a potential target gene of bta-miR-223 through comparison of the seed region of bta-miR-223 and the 3′ UTRs of potential target genes, which was conducted using TargetScan (Version 8.0). Design of forward and reverse primers for the RHOB 3′ UTR was performed using Primer Premier 5.0 (Table S1). Then, total RNA from bMECs was extracted using TRIzol Reagent (Invitrogen, Waltham, MA, USA), reverse transcribed to complementary DNA (cDNA) according to the product specification of the PrimeScript^TM^ RT reagent Kit with gDNA Eraser (Takara Biomedical Technology Co., Ltd., Beijing, China). The 3′ UTR fragment of RHOB was amplified according to the following reaction conditions: 20 μL total reaction volume consisting of forward/reverse primer 0.8 μL each; cDNA 1 μL; Taq PCR Master Mix 10 μL; ddH_2_O 7.4 μL, amplification result is in Figure S2. The 3′ UTR double-stranded cDNA fragment of RHOB was enzymatically cleaved and cloned into the XhoI-NotI site of the psiCHECK^TM^-2 vector (Promega Corp., Madison, WI, USA) containing the Renilla and firefly luciferase reporter genes, to form the psiCHECK-2-RHOB-wt (wild-type) dual-luciferase reporter gene recombinant vector. The psiCHECK-2-RHOB-wt recombinant plasmid was extracted with the Endo-Free plasmid Mini Kit (Omega Bio-Tek, Norcross, GA, USA). The resulting recombinant vector was verified by sequence analysis. The psiCHECK-2-RHOB-mut (mutant-type) was synthesized by Sangon Biotech (Shanghai) Co., Ltd., and the sequence was verified (Figure S3).

HEK-293T cells were seeded in 24-well plates at a density of 1.0 × 10^5^ cells/well [13]. After 24 h, the cells were transfected with the above plasmids using 2 μL of X-tremeGENE HP DNA transfection reagent (Roche, Penzberg, Germany) according to the manufactur-er’s instructions. The HEK-293 cells were co-transfected with psiCHECK-2-RHOB-wt recombinant plasmid (0.5 μg) and mimic of bta-miR-223 (also set control NC_mimic 0.5 μg, final working concentration 75 nM) [18]. After 48 h, Renilla luciferase activity (normalized to firefly luciferase activity) was assayed using a dual-luciferase reporter assay (Promega, Madison, WI) according to the manufacturer’s instructions. If the ratio of Renilla to firefly luciferase in the CHECK2 3′ UTR + mimic transfected group was more than 30% lower than that in the control group, it indicated that the miRNA interacted with the target gene 3′ UTR.

### 2.4. Transfection of bMECs

The bMECs were seeded into a 6-well plate and incubated until they reached 60–70% confluence, according to the cell transfection method we have previously reported [18]. Three replicates of each group were transfected with 5 μL of miR-223 mimic and NC_mimic, 10 μL of miR-223 inhibitor and NC_inhibitor, and 20 ng/μL of LPS was added at 42 h after transfection to stimulate inflammation in bMECs [19]. The bMECs were collected after 6 h for qPCR and Western blot analyses, and culture supernatants were collected for enzyme-linked immunosorbent assay (ELISA) analysis of inflammatory factors.

Transfection of siRNA-RHOB and NC was performed as described above. The bMECs were cultured for 42 h, when 20 ng/μL of LPS was added to initiate inflammatory responses. After 6 h, cells and supernatants were extracted for qPCR and ELISA, respectively.

### 2.5. RNA Isolation and Real-Time Quantitative PCR (qPCR)

In this experiment, total RNA was extracted from the above-transfected bMECs using TRIzol (Invitrogen, Waltham, MA, USA) according to the manufacturer’s protocol. The concentration and purity (OD260/280 ≥ 1.8; OD260/230 ≥ 1.0) of RNA was detected using a multifunctional microplate reader (Biotek Synergy SLXA, USA). Subsequently, the degradation and contamination of RNA were checked by agarose gel electrophoresis. Briefly, 1000 ng of qualified RNA was reverse transcribed into cDNA using a PrimeScript™ RT reagent Kit with gDNA Eraser (Takara Biomedical Technology Co., Ltd., Beijing, China). For bta-miR-223 detection, the RNA was reverse transcribed into a first-strand cDNA synthesis by the stem-loop method using PrimeScript™ RT reagent Kit with gDNA Eraser (Takara Biomedical Technology Co., Ltd., Beijing, China) and specific primer (5′ GTCGTATCCAGTGCAGGGTCCGAGGTATTCGCACTGGATACGA CTGGGGTAT 3′). Bta-miR-223 and all mRNA qPCR primers were designed using Primer Premier 5.0 (Table S1).

The qPCR reaction was performed using a 2 × M5 HiPer SYBR Premix Estaq (with Tli RNaseH) Kit (Mei5 Biotechnology Co., Ltd., Beijing, China) on a Bio-Rad CFX 96 Touch instrument (Bio-Rad, Hercules, CA, USA). The qPCR reaction mixture was 20 μL total volume, consisting of 2 × M5 HiPer SYBR Premix EsTaq (with Tli RNaseH) 10 μL, forward/reverse primers 0.8 μL each; cDNA 2 μL; ddH_2_O 6.4 μL. The qPCR thermocycling profile was as follows: pre-denaturation at 95 °C for 30 s, 95 °C for 5 s, 60 °C for 30 s, 45 cycles, 95 °C for 10 s, 65 °C for 5 s. Three replicate wells and 3 biological replicates were set up for each assay. After the qPCR was completed, the relative expression of genes was calculated using the 2^−ΔΔCt^ method using glyceraldehyde-3-phosphate dehydrogenase (GAPDH) and ribosomal protein S18 (RPS18) as internal references.

### 2.6. Western Blot Analysis

The bMECs were washed twice with PBS, then digested with trypsin into 1.5 mL centrifuge tubes. The digestion was terminated by adding an equal volume of medium, followed by centrifugation and discarding the supernatant. The cells were then washed with PBS and the supernatant was discarded following centrifugation. Total protein was extracted with via whole-cell Lysis Assay (Sangon Biotech Co. Ltd., Shanghai, China) and quantified with a BCA protein assay kit (KeyGEN BioTECH, Nanjing, China).

Equal amounts of total protein (40 μg) were extracted from each sample, resolved by SDS-PAGE with 12% (NF-κB and β-Actin) and 15% (RHOB), and transferred to nitrocellulose pre-membranes (GE Healthcare/Amersham, Pittsburgh, PA). The membranes were then blocked with 5% skim milk powder and then incubated overnight at 4 °C with an appropriate dilution of primary antibody. All of the primary and the secondary antibodies, including RHOB (1:200, catalog no. sc-8048), NF-κB (1:500, catalog no. AF5006), β-actin (1:500, catalog no. sc-47778), goat anti-mouse IgG-HRP (1:5000, catalog no. sc-17829), goat anti-rabbit IgG-HRP (1:5000, catalog no. sc-2030) and RHOB were purchased from Santa Cruz Biotechnology Inc. Then, goat anti-mouse IgG-HRP and goat anti-rabbit IgG-HRP were used to detect antigen bound primary antibodies. β-Actin was used as a control group for comparison between groups. Western Blot images were analyzed using Image J software.

### 2.7. ELISA for Cytokines

Following transfection and subsequent incubation for 48 h, the supernatants of the bMEC cultures from each treatment group were collected, and the secretion of cytokines IL-8, IL-6 and IL-1β was detected using the relevant ELISA kits (CUSABIO BIOTECH CO., Ltd., Wuhan, China) according to the manufacturer’s instructions.

### 2.8. Statistical Analysis

The experimental data are display as the mean ± standard error of the mean (SEM), with three replications. The significant differences between groups were tested by Student’s t-test using GraphPad Prism 9 (GraphPad Software, Inc., La Jolla, CA, USA). *p* < 0.05 indicates a significant difference between the treatment groups (LPS, mimic, inhibitor and siRNA-RHOB) and the control groups (control, NC_mimic, NC_inhibitor and NC).

## 3. Results

### 3.1. Phenotypic Verification of bMECs

**Figure 1:**
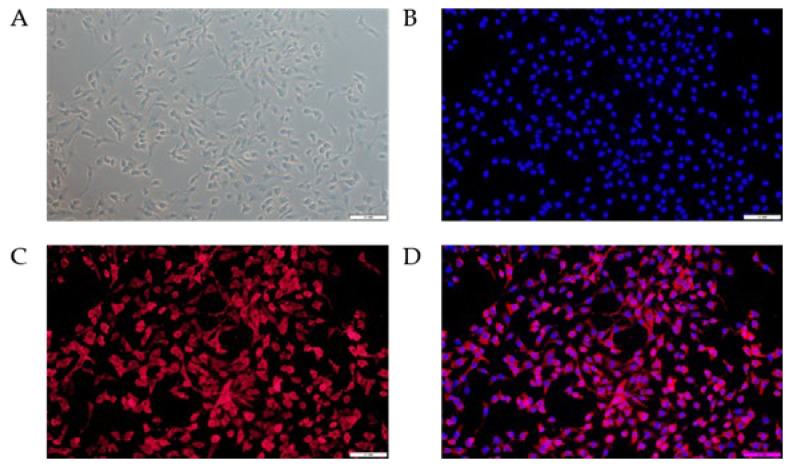
Phenotypic verification of bMECs by immunofluorescence with Cytokeratin 18 (200×). (**A**) Bright field. (**B**) DAPI staining. (**C**) Immunofluorescence with Cytokeratin 18 labeled with Cy3. (**D**) Merge of B and C.

The results of immunofluorescence staining showed that Cytokeratin 18 was positively expressed in the bMECs after subculturing and was mainly distributed in the cytoplasm (Figure 1). This observation suggests that the cultured bMECs in this study are pure and suitable for subsequent studies.

### 3.2. RHOB Is a Target Gene of bta-miR-223

**Figure 2:**
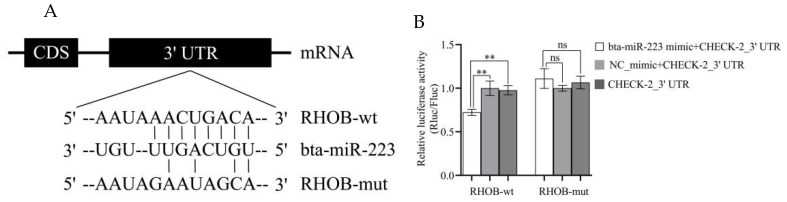
Bta-miR-223 directly targets the RHOB gene by binding to its 3′ UTR. Because RHOB-wt and RHOB-mut are the experimental results of two dishes of cells, the final data were normalized. (**A**) Predicted bta-miR-223 binding sites and experimentally introduced mutations within the RHOB 3′ UTR are shown. (**B**) Verification of the presence of the bta-miR-223 binding site in the RHOB 3′UTR by a dual-luciferase reporter assay. The experimental results are displayed as the mean ±SEM. ** *p* < 0.01, ns *p* > 0.05. CDS = coding Sequence; NC_mimic = negative control, compared with bta-miR-223 mimic; RHOB-wt = wild type of RHOB target site; RHOB-mut = RHOB sentinel mutation; CHECK-2_3′ UTR = dual-luciferase reporter recombinant vectors RHOB-wt-3′ UTR or RHOB-mut 3′ UTR and psi-CHECK-2; Rluc/Fluc = Renilla luciferase/firefly luciferase.

The 3′ UTR binding site of the RHOB gene by bta-miR-223 was predicted using TargetScan (Figure 2A). To verify that RHOB was a target gene of bta-miR-223, the gene’s 3′ UTR was ligated into the psiCHECK-2 vector (named RHOB-wt) and co-transfected with psiCHECK-2-RHOB-wt and bta-miR-223 mimic (negative control) into HEK-293 cells; the fluorescence efficiency was detected using a dual-luciferase reporter system. It was observed that the luciferase activity was significantly down-regulated in the co-transfected psiCHECK-2-RHOB-wt and bta-miR-223 mimic groups compared with the negative control (*p* < 0.001) (Figure 2B). In addition, when the 3′ UTR of the RHOB gene was mutated (RHOB-mut), the efficiency of bta-miR-223-mediated luciferase activity inhibition was found to be completely lost (*p* > 0.05) (Figure 2B). These results suggest that bta-miR-223 targets the 3′ UTR end of the RHOB gene, resulting in a reduction in luciferase activity.

### 3.3. The Expression of Bta-miR-223 Is Up-Regulated in Inflammatory bMECs

**Figure 3:**
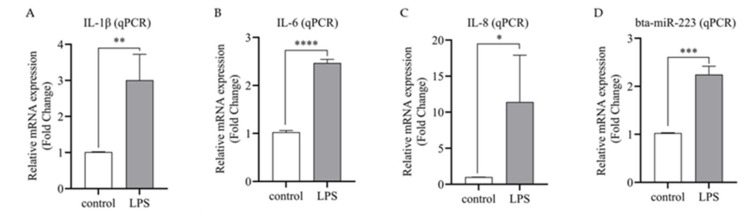
Establishment of inflammatory cell model and bta-miR-223 expression assay. (**A,B,C**)Expression of three inflammatory factors (IL-1β, IL-6 and IL-8) were significantly up-regulated in LPS-stimulated bMECs, indicating successful inflammatory cell model establishment. (**D**) Bta-miR-223 expression was significantly up-regulated in LPS-induced inflammation. The qPCR technique was used to detect the expression of each gene. The experimental results are displayed as the mean ±SEM. * *p* < 0.05, ** *p* < 0.01, *** *p* < 0.001, **** *p* < 0.0001.

To detect the expression of bta-miR-223 in inflammatory bMECs, the expression of bta-miR-223 after 6 h of LPS stimulation was analyzed by qPCR. It was observed that the expression of inflammatory factors IL-1β, IL-8 and IL-6 were significantly up-regulated (*p* < 0.01) in LPS-stimulated bMECs (Figure 3A–C). Therefore, the model of inflammatory bMECs was successfully established. Subsequently, the expression of bta-miR-223 was significantly up-regulated (*p* < 0.01) in the inflammatory cell model (Figure 3D), suggesting that bta-miR-223 may play some role in the inflammatory response of bMECs, which requires further validation.

### 3.4. Bta-miR-223 Inhibits the Expression of RHOB and NF-κB Genes

**Figure 4:**
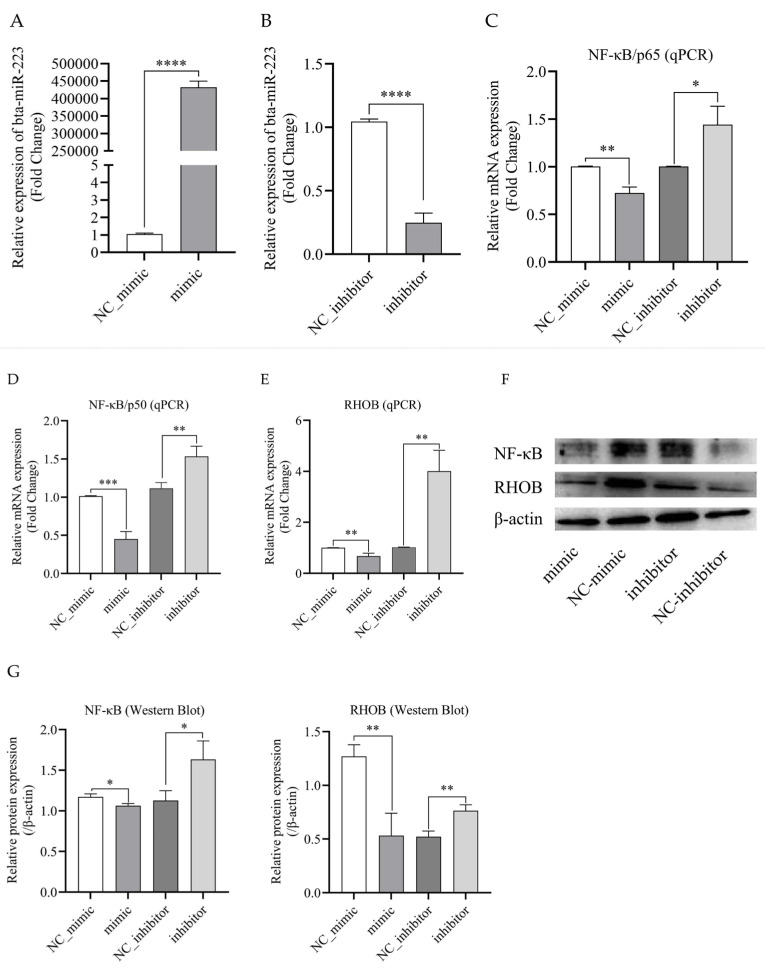
Bta-miR-223 inhibits the expression of RHOB and NF-κB in bMECs. The bMECs were transfected with bta-miR-223 mimic or NC_mimic, or either inhibitor or NC_inhibitor. Then, 48 h after transfection, the expression levels of genes and proteins were detected by performing qPCR and Western blot, respectively. (**A**) The transfection efficiency of bta-miR-223 mimic was detected by performing qPCR. (**B**) The transfection efficiency of bta-miR-223 inhibitor was detected by performing qPCR. (**C**) qPCR analysis of NF-κB/p65 in bMECs transfected with bta-miR-223 mimic or inhibitor. (**D**) qPCR analysis of NF-κB/p50 in bMECs transfected with bta-miR-223 mimic or inhibitor. (**E**) qPCR analysis of RHOB in bMECs transfected with bta-miR-223 mimic or inhibitor. (**F**,**G**) Western blot analysis of NF-κB and RHOB protein level expressions in bMECs transfected with bta-miR-223 mimic or inhibitor. Western blot images of NF-κB and RHOB from the same total protein were analyzed using Image J software. The density quantification of Western blot was normalized to that of the same batch β-actin in order to perform a comparative analysis. The experimental results are displayed as the mean ± SEM. * *p* < 0.05, ** *p* < 0.01, *** *p* < 0.001, **** *p* < 0.0001.

To explore the function and molecular mechanism of bta-miR-223 in bMECs, the cells were transfected with miR-223 mimic, NC_mimic, miR-223 inhibitor, or NC_inhibitor. The qPCR results show that, in bMECs, bta-miR-223 mimics significantly increased the expression of mature bta-miR-223 (*p* < 0.0001) (Figure 4A), whereas bta-miR-223 inhibitors significantly decreased the expression of mature bta-miR-223 (*p* < 0.001) (Figure 4B). Next, mRNA and protein levels of RHOB and NF-κB in the bta-miR-223 mimic transfection group and inhibitor transfection group, as well as their respective control groups, were analyzed by qPCR and Western Blot, respectively. The results indicated that compared with the negative control (NC_mimic), overexpression of bta-miR-223 in bMECs significantly suppressed the mRNA and protein expression levels of RHOB and NF-κB (*p* < 0.05) (Figure 4C–G). Conversely, inhibition of bta-miR-223 significantly up-regulated the mRNA and protein expression (*p* < 0.05) (Figure 4C–G). These results suggest that bta-miR-223 inhibits the expression of RHOB and NF-κB at the transcriptional and translational levels.

### 3.5. Bta-miR-223 Negatively Regulates the Expression and Secretion of IL-1β, IL-6 and IL-8 in LPS-Stimulated bMECs

**Figure 5:**
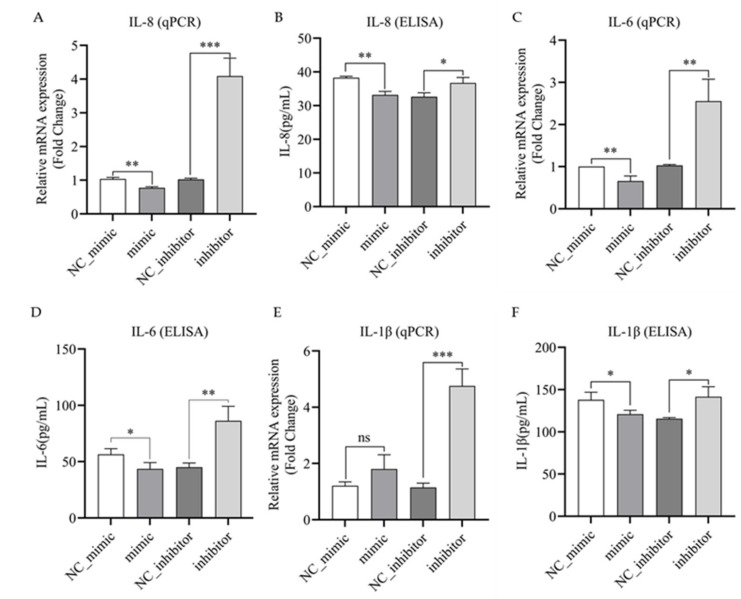
Bta-miR-223 inhibits the production of IL-1β, IL-8 and IL-6 in bMECs. The bMECs were transfected with bta-miR-223 mimic or negative control (NC_mimic) and bta-miR-223 inhibitor or negative control (NC_inhibitor). bMECs were stimulated with LPS for 6 h. Then the cells were collected, and the expression levels of IL-8 (**A**,**B**), IL-6 (**C**,**D**) and IL-1β (**E**,**F**) mRNA and secretion levels of inflammatory factors were detected. qPCR and ELISA were used to detect the expression of inflammatory factors in each group of cells at the mRNA and secretory levels, respectively. The experimental results are displayed as the mean ±SEM. ns *p* > 0.05, * *p* < 0.05, ** *p* < 0.01, *** *p* < 0.001.

To investigate whether bta-miR-223 is involved in regulating the expression of inflammatory factors in bMECs, the mimic and inhibitor of bta-miR-223 and their NCs were transfected into bMECs. The bMECs were then stimulated with LPS to produce an inflammatory response, and the mRNA expression of IL-1β, IL-8 and IL-6 and the secretion levels of inflammatory factors were detected using qPCR and ELISA, respectively. The results showed that compared with the control group (NC_mimic), the bta-miR-223 mimic group significantly inhibited the expression of IL-8 and IL-6 mRNA and protein (*p* < 0.05) (Figure 5A–D). Although IL-1β mRNA was not significantly inhibited (*p* > 0.05), protein secretion was significantly inhibited (*p* < 0.05) (Figure 5E,F). Meanwhile, the bta-miR-223 inhibitor group significantly up-regulated the expression of inflammatory factors IL-8, IL-6 and IL-1β at the mRNA and secretory levels compared with the control group (NC_inhibitor) (*p* < 0.05) (Figure 5A–F). The above results indicate that bta-miR-223 inhibits the expression of IL-8 and IL-6 transcription and secretion in bMECs, whereas IL-1β is inhibited by bta-miR-223 only at the secretion level. Taken together, bta-miR-223 is a negative regulator of inflammatory responses in bMECs.

### 3.6. Silencing of RHOB Inhibits the Expression and Secretion of IL-1β, IL-6 and IL-8 in LPS-Induced bMECs

**Figure 6:**
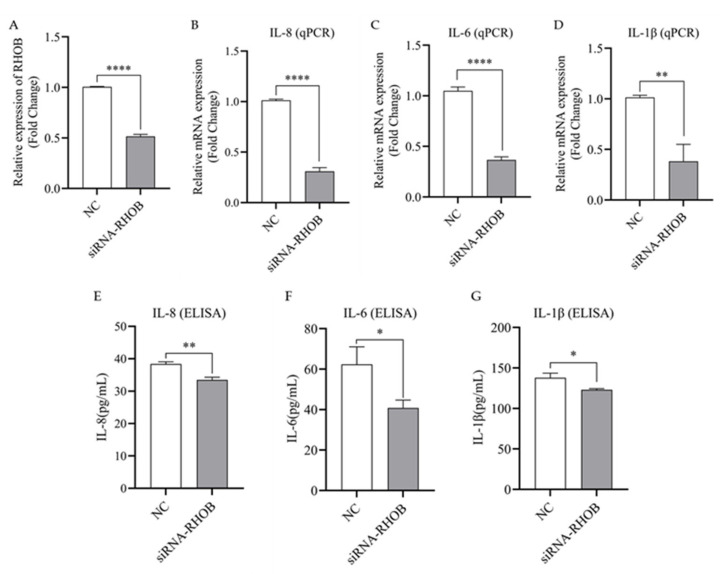
Expression changes of inflammatory factors after silencing of target gene RHOB. (**A**) RHOB silencing efficiency assay: cells were then collected and the expression of IL-8 (**B**,**E**), IL-6 (**C**,**F**) and IL-1β (**D**,**G**) at mRNA and secreted protein levels were examined. The bMECs were transfected with RHOB small interfering fragments. bMECs were stimulated with LPS for 6 h after transfection for 42 h. The expression of inflammatory factors at the mRNA and secreted protein levels in each group of cells were measured by qPCR and ELISA, respectively. The experimental results are displayed as the mean ±SEM. * *p* < 0.05, ** *p* < 0.01, **** *p* < 0.0001.

To further confirm that bta-miR-223 inhibits the expression of inflammatory factors at the mRNA and secretion levels by regulating RHOB, an interference test of RHOB in bMECs was conducted and analyzed by qPCR and ELISA to detect inflammatory markers before and after silencing RHOB in bMECs. The results show that transfection of siRNA-RHOB resulted in a 50.1% down-regulation of RHOB gene expression in bMECs (*p* < 0.0001, Figure 6A). Silencing of RHOB significantly inhibited the expression of IL-8, IL-6 and IL-1β gene expression (*p* < 0.01, Figure 6B–D), and also significantly suppressed the secretion of the respective proteins in bMECs (*p* < 0.05, Figure 6E–G). Therefore, the above results indicate that silencing RHOB inhibits the release of inflammatory factors in bMECs, which is consistent with the results of the bta-miR-223 mimic group, resulting in the attenuation of the inflammatory response in bMECs.

## 4. Discussion

miRNAs are important regulators of several components of mammalian immune responses. It has been demonstrated previously that miR-223 is significantly up-regulated in inflammation-induced granulocytes, dendritic cells, T cells, endothelial cells and epithelial cells [20]. One study showed that bta-miR-223 was up-regulated approximately 2.5- to 3-fold in dairy cows with mastitis [21,22]. In the present experiment, it was observed that the expression of bta-miR-223 was also up-regulated in bMECs during inflammation, which corroborates the above findings. Regulation of gene expression by miRNA occurs through specific binding to target genes containing complementary nucleotide sequences. The bovine and human miR-223 sequences are identical. The bovine and human RHOB sequences are highly homologous, however, they are not identical. Although miR-223 has been identified to regulate RHOB gene expression in humans, the molecular regulatory mechanism in LPS-induced mastitis in cows has not been examined. It has been shown that in human lung inflammation, miR-223 can alleviate the inflammation caused by the release of inflammatory factors in the lung by directly targeting the 3′ UTR of RHOB, and low expression of RHOB has been closely associated with a lack of NF-κB signaling pathway activity [11]. In the present experiments, the bta-miR-223 binding site within the 3′ UTR of the RHOB gene was predicted and verified by a dual-luciferase reporter assay. It was observed that bta-miR-223 does indeed bind to the RHOB target, which is consistent with the findings of the above-mentioned authors.

RHOB is a low molecular weight GTPase [23]. In human colon epithelial cells, it was reported that miR-223 targeting of RHOB limited the spread of colon cancer cells [24]. In non-small cell lung adenocarcinoma (NSCLC) cells, miR-223 mediated RHOB knockdown and significantly inhibited RHOB protein expression, thereby suppressing the development of NSCLC [14]. In dendritic cells, miR-223 down-regulated RHOB protein expression, thereby promoting T cell differentiation [15]. In human acute lung injury (ALI), the activation of RHOB potentiates the TLR4/NF-κB signaling pathway activity, resulting in the release of inflammatory factors [11]. Interestingly, in LPS-induced inflammation in mouse macrophages, miR-223 inhibited the release of several inflammatory factors (tumor necrosis factor-α (TNF-α), IL-6 and IL-1β) associated with TLR4 activation by targeting RHOB, thereby causing macrophage inflammatory damage [16]. Meanwhile, studies have reported that RHOB is a negative regulator of the NF-κB (p65/50) signaling pathway, which has been demonstrated to act in a dose-dependent manner [25,26]. This resulted in reduced expression of inflammatory factors, thereby alleviating the inflammatory damage induced by LPS [27]. In macrophages, the expression of inflammatory factors (TNFα, IL-6 and IL-1β) can be significantly inhibited at the mRNA and protein levels by overexpression of RHOB [28]. In contrast, the silencing of RHOB in human endothelial cells inhibited the production of IL-8 and IL-6 [29]. In this experiment, inflammatory factors (IL-6, IL-8 and IL-1β) were found to be suppressed at both mRNA and protein levels when RHOB expression was knocked down, which is consistent with the above results. Therefore, RHOB is a potential novel target for the treatment of mastitis in cows.

**Figure 7:**
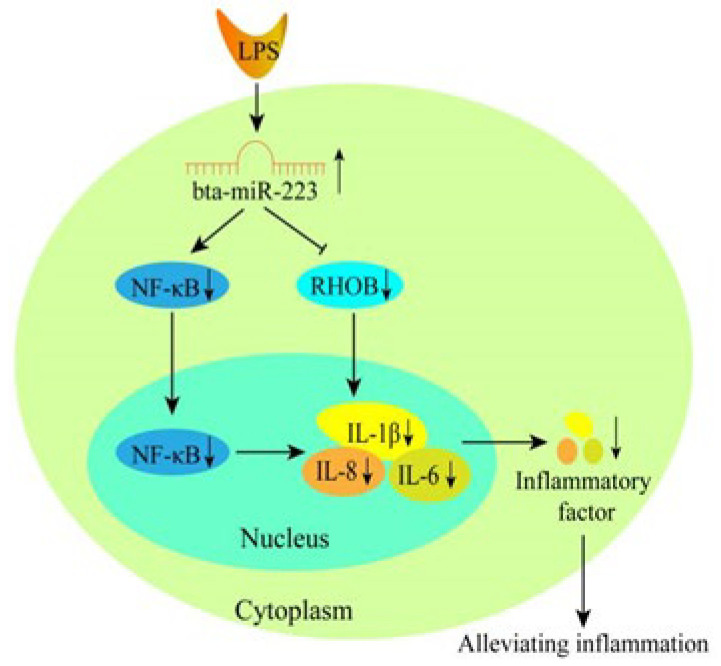
Summary of the mechanism of action of bta-miR-223 in bMEC inflammation. In the LPS-stimulated bMEC response, high expression of bta-miR-223 suppresses the expression of its target genes, RHOB and NF-κB at the protein level, ultimately leading to inhibition of the inflammatory factors IL-1β, IL-8 and IL-6 at the transcriptional and translational levels, thereby alleviating the inflammatory response in bMECs.

Once pathogenic bacteria have entered the breast, bMECs respond rapidly to initiate the antimicrobial response [30,31]. Interestingly, mRNA levels of all inflammatory factors increased rapidly 2–4 h after stimulation of bMECs with LPS and LTA, but only LPS stimulation resulted in sustained inflammatory factor expression [19,32] of the TLR4/NF-κB inflammatory signaling pathway [33]. It is therefore hypothesized that the NF-κB signaling pathway is only continuously activated in LPS-stimulated bMECs. According to Han et al. [9], bta-miR-223 in LTA-induced mastitis does not inhibit the precursor protein of NF-κB (p50/p65) and only inhibits phosphorylation of NF-κB. In contrast, in LPS-induced bMECs, overexpression of bta-miR-146a inhibited the precursor protein of NF-κB [18]. In addition, it has been shown that heat-inactivated LPS activates NF-κB through the TLR4/NF-κB signaling pathway [33,34]. In response to activation of the TLR signaling pathway, NF-κB binds to the promoters of inflammatory factor genes, resulting in increased mRNA and protein levels of the inflammatory cytokines TNFα, IL-6, IL-1β and IL-8 within 1–3 h of LPS activation [35,36,37]. It is well established that IL-1β and IL-8 play an important role in the recruitment and activation of immune cells via local and systemic effects to eliminate pathogens, thereby inhibiting the development of acute inflammation [38,39,40]. IL-6 is the main pro-inflammatory cytokine mediating the inflammatory response and plays an important role in the acute phase response to pathogen detection [41]. In order to investigate whether bta-miR-223 is involved in regulating the expression of inflammatory cytokines in cows, LPS-stimulated bMECs were employed as a model of inflammation. The results showed that overexpression of bta-miR-223 could down-regulate the translation of RHOB, NF-κB and the associated inflammatory factors (IL-8, IL-6 and IL-1β). Silencing RHOB also effectively suppressed the secretion of inflammatory factors, further indicating that bta-miR-223 alleviated the inflammation of bMECs caused by LPS via suppression of NF-κB and RHOB (Figure 7). Taken together, these data implicate a potential mechanism of action and the utility of bta-miR-223 in the molecular diagnosis of mastitis in dairy cows.

## 5. Conclusions

In this study, it was demonstrated that elevated expression of bta-miR-223 in LPS-induced bMECs suppressed the expression of the RHOB and NF-κB genes, thus resulting in decreased secretion of inflammatory mediators IL-1β, IL-8 and IL-6 (Figure 7). Furthermore, the results of inflammatory factor secretion from bMECs after silencing the RHOB gene corroborated the observations from bta-miR-223 overexpression experiments. The above results suggest that bta-miR-223 is a negative regulator of inflammation in bMECs and can alleviate the inflammatory response of bMECs by knockdown of RHOB gene expression. This study sheds light on the regulatory mechanism of bta-miR-223 on the immune response to mammary gland infection in cows at the cellular level, providing a new molecular target for the treatment of mastitis.

## Data Availability

Data is contained within the article and Supplementary Materials.

## Supplementary Materials

The following supporting information can be downloaded at: https://www.mdpi.com/2073-4409/11/19/3144/s1, Figure S1A,B: Transfection efficiency detection of miR-223 in bMECs; Figure S1C: Transfection efficiency detection of RHOB in bMECs; Figure S2: Gel electrophoresis result of RHOB amplification; Figure S3: Sequencing results of RHOB; Table S1: The list of all primer sequences.
