# Correction: Jiao et al. Bta-miR-223 Targeting the RHOB Gene in Dairy Cows Attenuates LPS-Induced Inflammatory Responses in Mammary Epithelial Cells. *Cells* 2022, *11*, 3144

**DOI:** 10.3390/cells12010165

**Published:** 2022-12-30

**Authors:** Peng Jiao, Jinpeng Wang, Jian Yang, Xingping Wang, Zhuoma Luoreng

**Affiliations:** 1 School of Agriculture, Ningxia University, Yinchuan 750021, China; 2 Key Laboratory of Ruminant Molecular Cell Breeding, Ningxia Hui Autonomous Region, Yinchuan 750021, China

In the original publication [1], the pictures of Figure 4 are correct, however, there were some errors in the legend (C)/(D)/(E)/(F) and (G) of Figure 4 due to the author’s negligence. 

The CORRECT legend of Figure 4 in the original publication [1] is as follows:

**Figure 4.** Bta-miR-223 inhibits the expression of RHOB and NF-κB in bMECs. The bMECs were transfected with bta-miR-223 mimic or NC_mimic, or either inhibitor or NC_inhibitor. Then, 48 h after transfection, the expression levels of genes and proteins were detected by performing qPCR and Western blot, respectively. (A) The transfection efficiency of bta-miR-223 mimic was detected by performing qPCR. (B) The transfection efficiency of bta-miR-223 inhibitor was detected by performing qPCR. (C) qPCR analysis of NF-κB/p65 in bMECs transfected with bta-miR-223 mimic or inhibitor. (D) qPCR analysis of NF-κB/p50 in bMECs transfected with bta-miR-223 mimic or inhibitor. (E) qPCR analysis of RHOB in bMECs transfected with bta-miR-223 mimic or inhibitor. (F, G) Western blot analysis of NF-κB and RHOB protein level expressions in bMECs transfected with bta-miR-223 mimic or inhibitor. Western blot images of NF-κB and RHOB from the same total protein were analyzed using Image J software. The density quantification of Western blot was normalized to that of the same batch β-actin in order to perform a comparative analysis. The experimental results are displayed as the mean ± SEM. * *p* < 0.05, ** *p* < 0.01, *** *p* < 0.001, **** *p* < 0.0001.

The authors state that the scientific conclusions are unaffected. This correction was approved by the Academic Editor. The original publication has also been updated.
