# Supplementary material for: Bta-miR-223 Targeting the RHOB Gene in Dairy Cows Attenuates LPS-Induced Inflammatory Responses in Mammary Epithelial Cells
Source: Cells. 2022 Oct 6;11(19):3144. doi: 10.3390/cells11193144 (PMC9563457; doi:10.3390/cells11193144)
Supplement: Supplementary file 1 [file cells-11-03144-s001.zip › Supplementary Materials/Figure S1 A-B Transfection efficiency detection of miR-223 in bMECs - ╕▒▒╛.pdf]

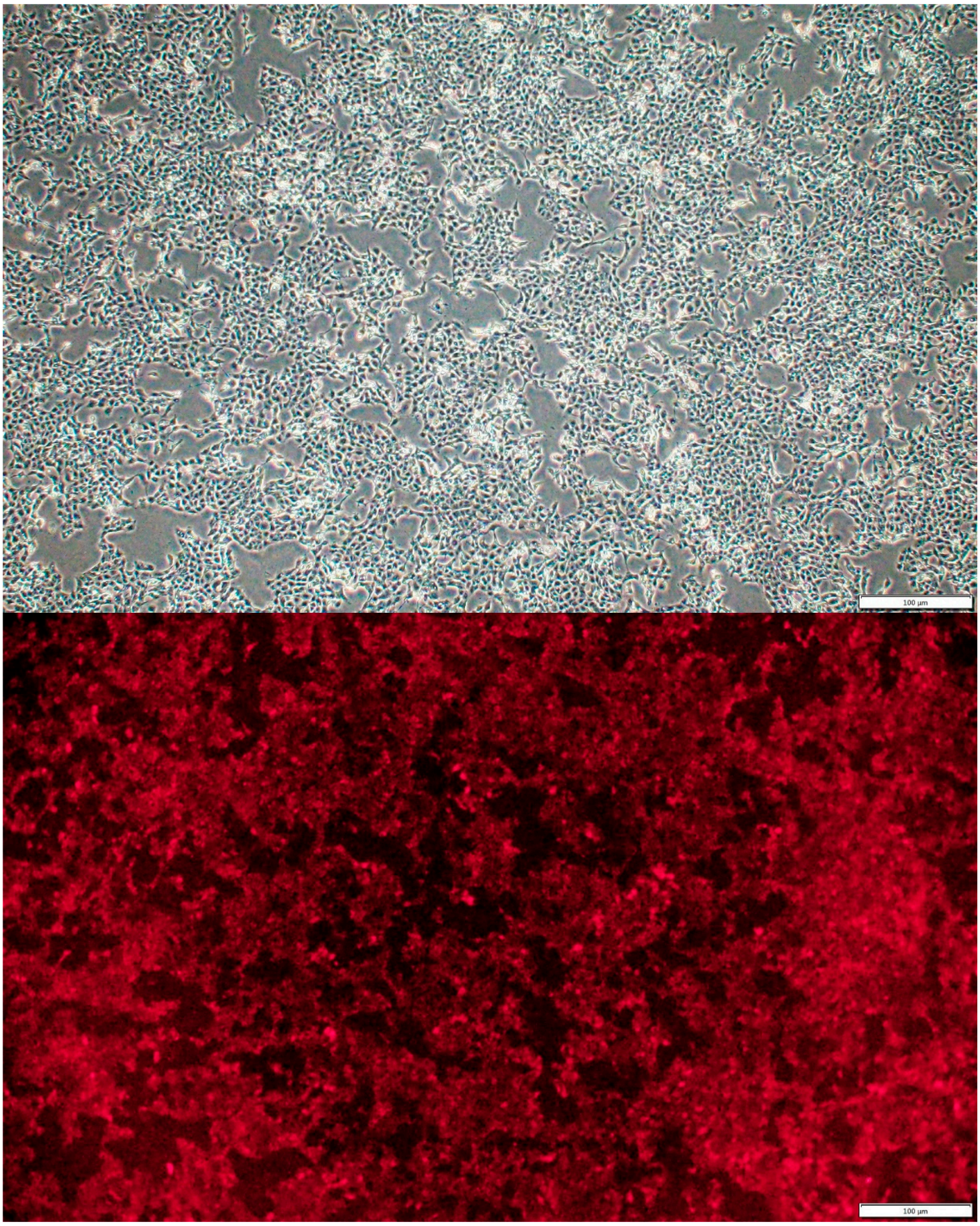

Figure S1 A. Cy3 labeled NC fluorescence transfection efficiency

Note: Magnification 40

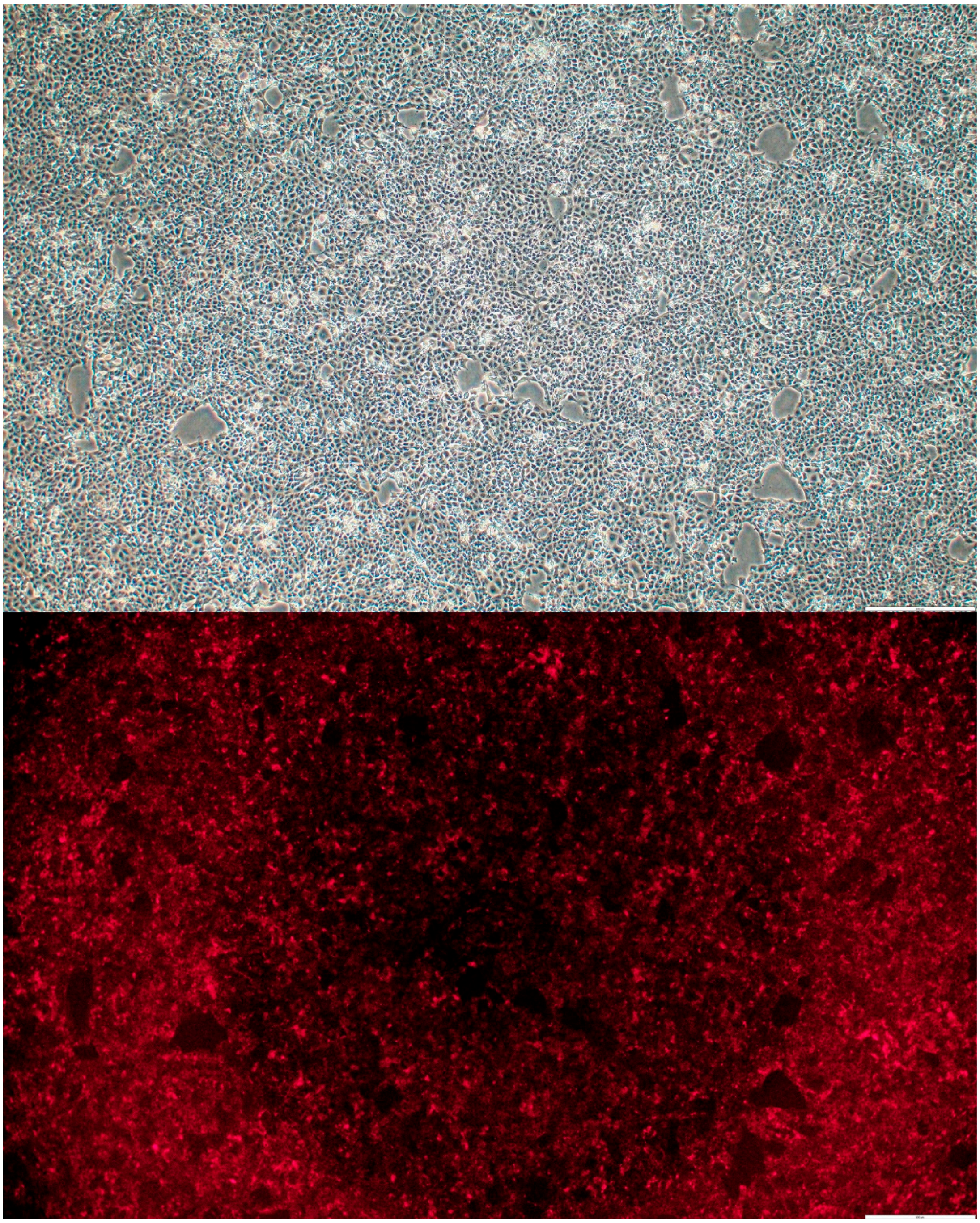

Figure S1 B. Cy3 labeled NC fluorescence transfection efficiency

Note: Magnification 40
