# Supplementary material for: Bta-miR-223 Targeting the RHOB Gene in Dairy Cows Attenuates LPS-Induced Inflammatory Responses in Mammary Epithelial Cells
Source: Cells. 2022 Oct 6;11(19):3144. doi: 10.3390/cells11193144 (PMC9563457; doi:10.3390/cells11193144)
Supplement: Supplementary file 1 [file cells-11-03144-s001.zip › Supplementary Materials/Figure S3 Sequencing results of RHOB.pdf]

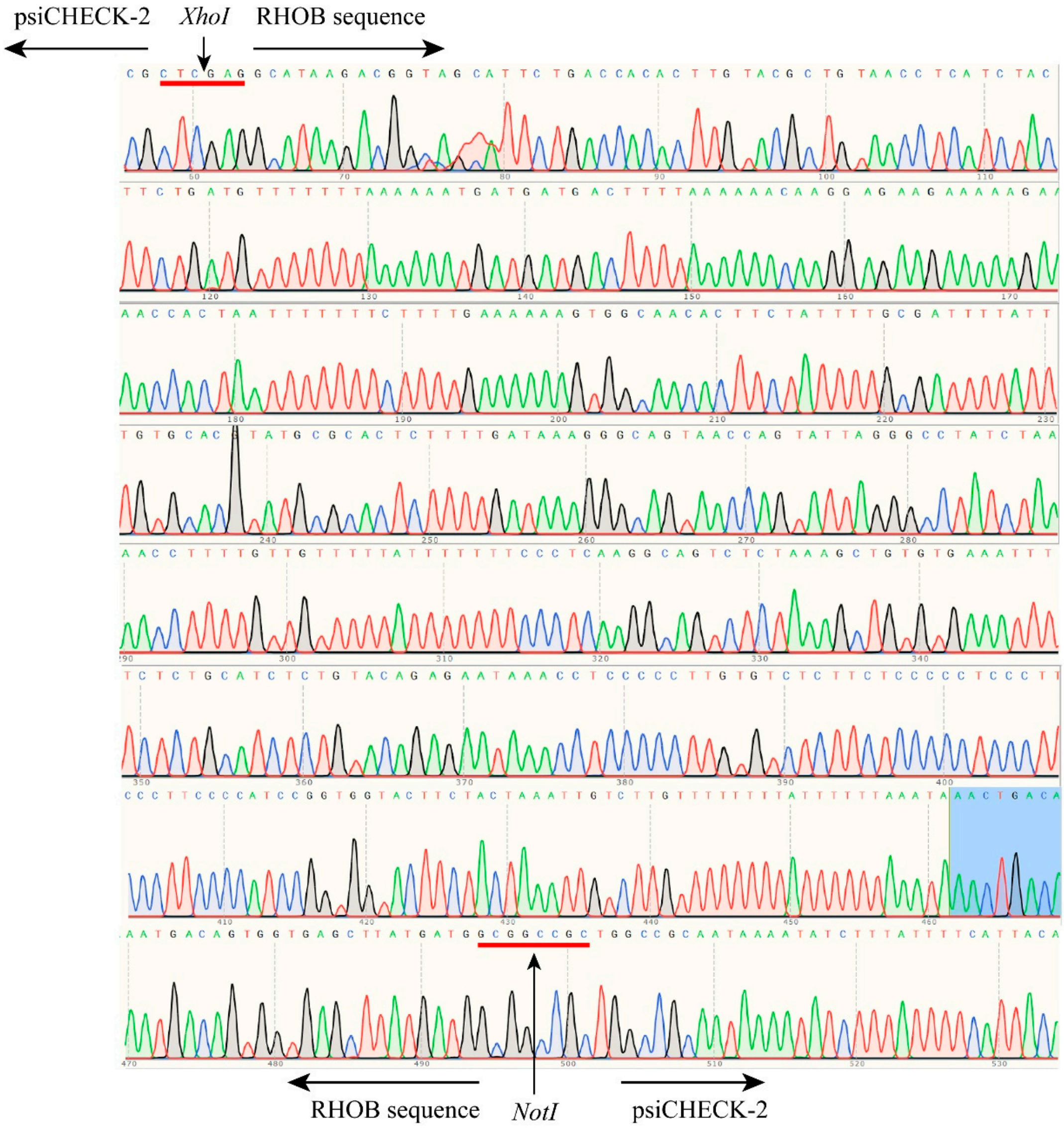

Figure S3 A. Sequencing results of RHOB-wt
(The blue background is the binding site with bta-miR-223)

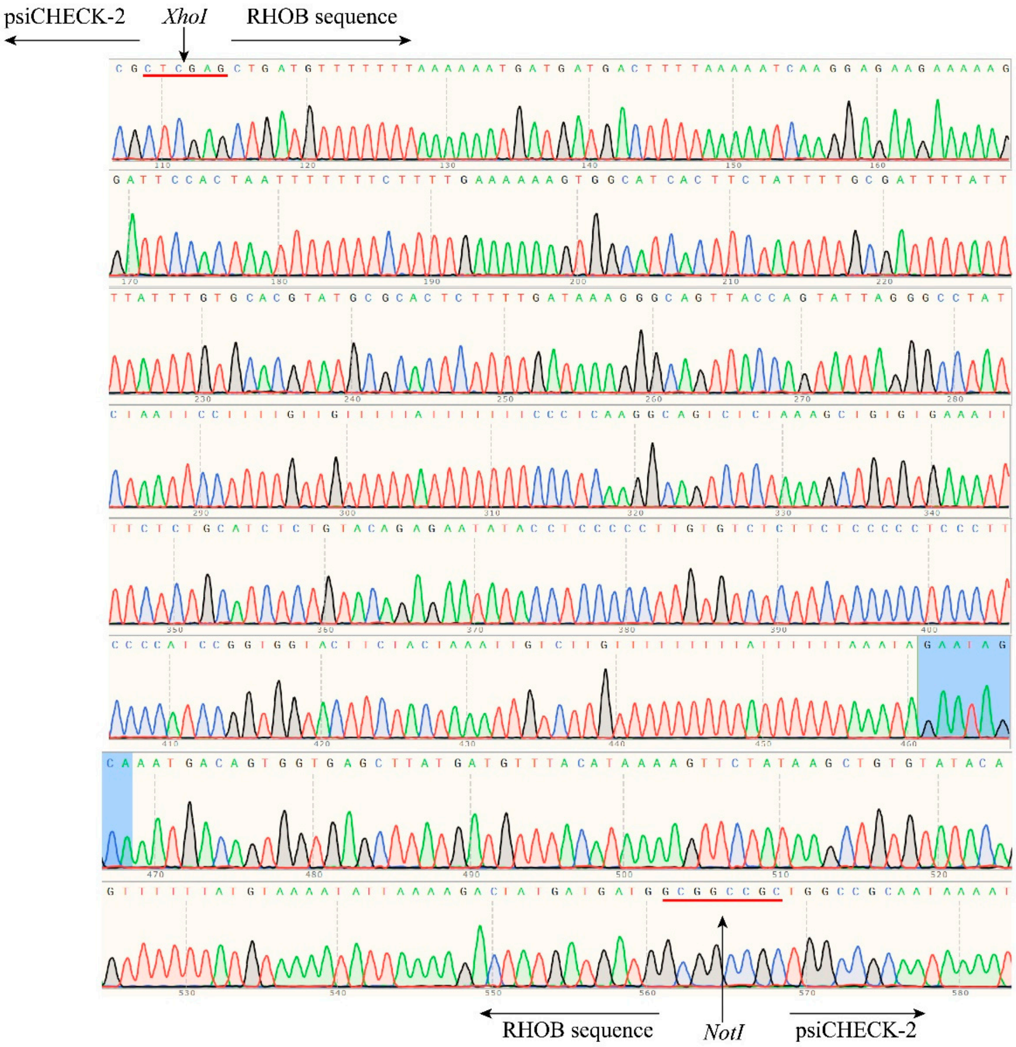

Figure S3 B. Sequencing results of RHOB-mut
(The blue background is mutation site)
