# Supplementary figures and images for: Bta-miR-223 Targeting the RHOB Gene in Dairy Cows Attenuates LPS-Induced Inflammatory Responses in Mammary Epithelial Cells
Source: Cells. 2022 Oct 6;11(19):3144. doi: 10.3390/cells11193144 (PMC9563457; doi:10.3390/cells11193144)

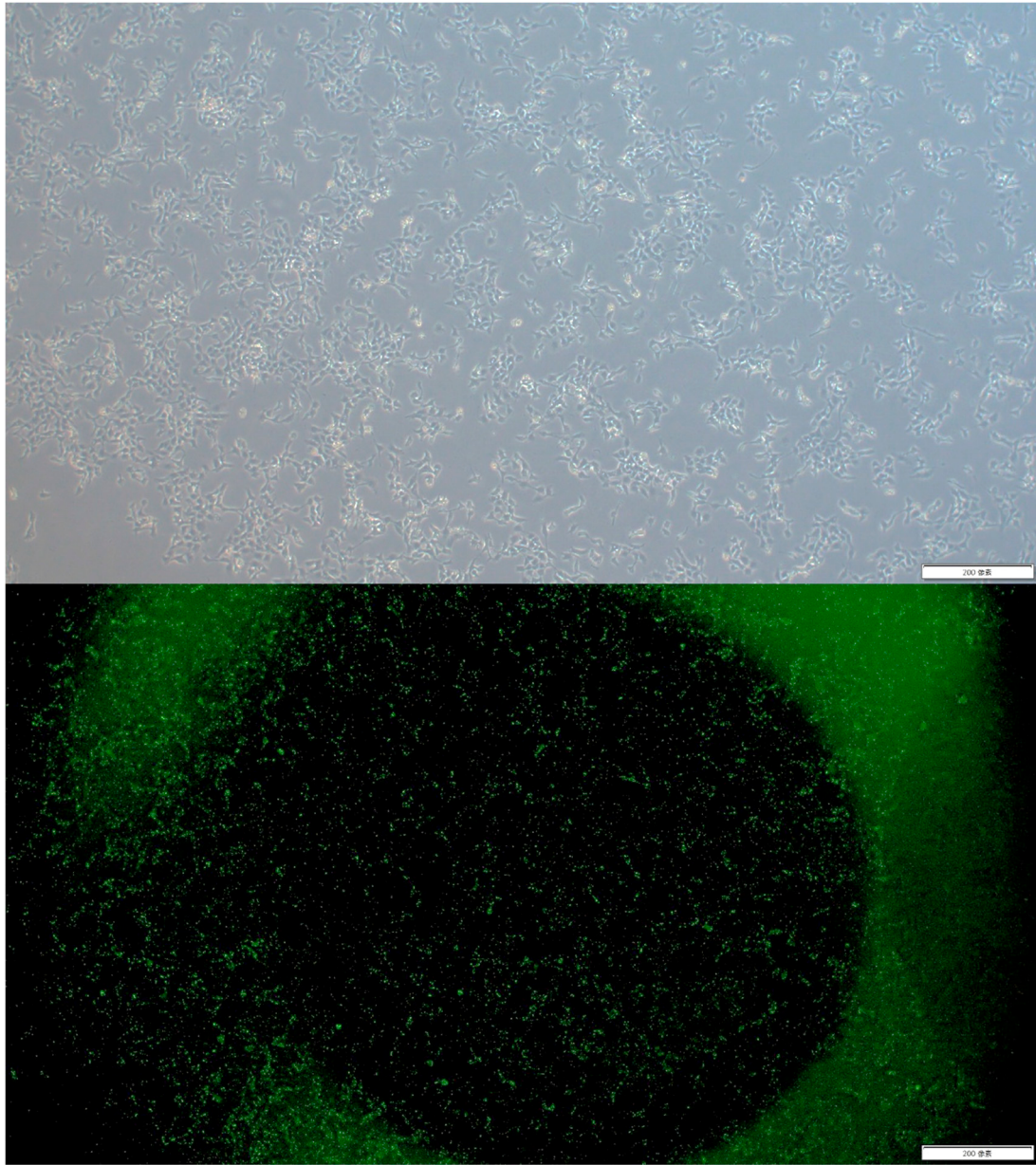

Figure S1 C. FAM labeled NC fluorescence transfection efficiency

Note: Magnification 40

Supplement: Supplementary file 1 [file cells-11-03144-s001.zip › Supplementary Materials/Figure S1 C Transfection efficiency detection of RHOB in bMECs.pdf]

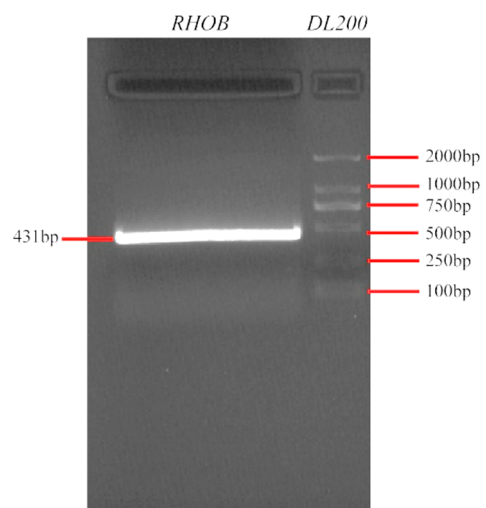

Figure S2: Gel electrophoresis result of

Supplement: Supplementary file 1 [file cells-11-03144-s001.zip › Supplementary Materials/Figure S2 Gel electrophoresis result of RHOB amplification.pdf]
